# “Lubricant” or “Stumbling Block”?: The Paradoxical Association Between Team Authoritarian Leadership and Creative Deviance

**DOI:** 10.3389/fpsyg.2022.835970

**Published:** 2022-03-31

**Authors:** Jing Xu, Yong-Zhou Li, De-Qun Zhu, Jing-Zhi Li

**Affiliations:** ^1^Evergrande School of Management, Wuhan University of Science and Technology, Wuhan, China; ^2^School of Economics and Management, Shangrao Normal University, Shangrao, China; ^3^School of Labor Relations and Personnel, Renmin University of China, Beijing, China

**Keywords:** team authoritarian leadership, creative deviance, dual occupational stress, prevention regulatory focus, individual mindfulness

## Abstract

Recently, creative deviance has been lauded to be an innovation-enhancing approach with applications in many new and high-tech domains. Previous study on antecedents to creative deviance remains scattered and vague. Our research conceptualizes creative deviance from the perspective of independent innovation and explores its antecedents, mechanisms, as well as conditions. Team authoritarian leadership is conceptualized as a contradictory unity as it mixes advantages and disadvantages. However, it is surprising to find that there are very few researches that have examined its relevant influence mechanisms and boundary conditions for authoritarian leadership. Contributing to an advanced understanding of authoritarian leadership in research and development teams, we investigated whether team authoritarian leadership is positively or negatively related to creative deviance. Drawing on social information processing theory and regulatory focus theory, we supposed that team authoritarian leadership facilitates creative deviance when the degree is low and inhibits it when the degree is high; dual occupational stress and prevention regulatory focus play mediation roles between team authoritarian leadership and creative deviance respectively, both variables play a chain mediation role in that relationship; and the mindfulness characteristic of an individual moderates the inverted-U team authoritarian leadership-creative deviance association, such that this association is weaker with low individual mindfulness. With two-phase questionnaire data collected from 433 members in 82 R&D teams of high-tech enterprises in electronic information technology, new material technology, new medical technology, resource and environment technology and advanced manufacturing technology randomly selected from five provinces in eastern China, these hypotheses are supported empirically. Overall, we find that, our study broadens antecedents and the relevant occurrence mechanisms of creative deviance when studied through a leadership management lens. Moreover, our research enriches the cognate studies on authoritarian leadership by empirically demonstrating that team authoritarian leadership may function as an double-edged sword of creative deviance in the R&D workplace. These above findings offer insightful thoughts to scholars in the field of authoritarian leadership and bring practical suggestions for team superiors who seek to implement best innovation practice.

## Introduction

*Xiaochuan Wang insisted on the development of Sogou browser against the background of strong opposition from the senior management, and finally achieved a “myth” with a market value of $35 billion.*

*-https://www.sohu.com/a/161478919_172964*

*The creator of Pontiac’ s successful Fiero model was repeatedly compelled to stop working; the designer of HP’ s highly profitable electrostatic displays was ordered by David Packard himself to stop the project immediately.*

*-from Mainemelis’ s research in 2010*

The aforesaid are interesting issues. In the era when high-tech enterprises increasingly face a complex environment, R&D employees’ agility and flexibility become more essential to innovation competitiveness than ever before. Recently, innovation researchers emphasized an emerging phenomenon, where innovational activities are guided by the pro-organization vision, rather than exclusively by those in higher leadership positions (Oettingen et al., 2001; Zhang and Arvey, 2009; Spagnoli et al., 2020). Scholars have realized that R&D employees’ resistance toward formal norms and rules is not necessarily a terrible thing—sometimes, it can build a powerful basis for long-term improvement instead (Vadera et al., 2013; Criscuolo et al., 2014). For example, R&D personnel, e.g., Xiaochuan Wang, who had adopted unconventional ways of innovating by deviating from universal ways of working (such as functional disobedience) were found to be able to implement pioneering ideas in terms of ignoring managerial instructions selectively (Criscuolo et al., 2014). Mainemelis (2010) and Lin et al. (2016) named this innovative behavior that violates referent norms to benefit the organization “creative deviance.” However, few studies explored the antecedents and occurrence mechanisms of creative deviance from the leadership perspective (Lin et al., 2016; Javed et al., 2017). Conceptually, authoritarian leadership is a power-centric phenomenon (Farh and Cheng, 2000) whereby leaders “provide a clear, unambiguous, and direct prototype” (Wang and Guan, 2018, p. 357), and “centralize decision-making” (Spagnoli et al., 2020, p. 620310). Given the controlling nature of this construct (Farh and Cheng, 2000; Cheng et al., 2004), we focus on one typical antecedent of creative deviance through the lens of breaking controlling shackle—how team authoritarian leadership influences the emergence of creative deviance. The team authoritarian leadership–creative deviance relationship exhibits a dynamic influence process among authoritarian superiors and subordinates whose objective is to foster team achievement or product development or both. However, our understanding of this theme is still limited in at least three fundamental aspects.

First, leadership scholars have worked to assess what influences authoritarian leadership brings to organizations. Table 1 reveals the details of relevant studies visually. Many empirical studies have demonstrated that authoritarian leadership, as “toxic” leadership (Spagnoli et al., 2020), yields harmful outcomes (Chen and Kao, 2009; Chan et al., 2013; Chen et al., 2014; Dedahanov et al., 2016; Rui and Qi, 2021). However, we should caution that these are not always the truths. Authoritarian leadership is also confirmed to be positively related to employee responses (Cheng et al., 2004), subordinates’ welfare (Aycan, 2006), subsequent revenue growth (Huang et al., 2015), procedural/interactional fairness perception and tacit knowledge sharing intention (Chen et al., 2018), learning goal orientation (Wang and Guan, 2018), quality of communication (Karakitapoglu-Aygün et al., 2021). These conclusions suggest the need for multidimensional perspectives on the impacts from authoritarian leadership. Therefore, the first purpose of our research is to examine the team authoritarian leadership–creative deviance relationship.

**TABLE 1:** Theories and impacts of authoritarian leadership.

Researchers	Samples	Contexts	Theories	Dependent variables
Cheng et al., 2004	543 low- to mid-level managers and staff	60 Taiwanese enterprises	Paternalistic leadership theory	Subordinate responses (positive relationship)
Aycan, 2006	First phase, 60 employees; second phase, 177 employees; third phase, 100 employees	First phase, private and public sector organizations in Turkey; second phase, private sector; third phase, a large privately owned rubber factory	PM leadership theory	Subordinates’ welfare (positive relationship); organizational commitment (negative relationship)
Chen and Kao, 2009	60 subordinates supervised by 52 Chinese expatriate managers from the company’s 31 overseas branches	Chinese MNEs based in Taiwan that operate 31 branches in Asia, Europe, America, and Oceania	Subjective well-being theory	Non-Chinese subordinates’ psychological health (negative relationship)
Zhang et al., 2011	163 work groups involving 973 employees	Twelve Chinese companies	Traditional Chinese leadership theory	Group creativity (negative relationship); collective efficacy (negative relationship); knowledge sharing (negative relationship)
Chan et al., 2013	686 immediate supervisor–subordinate (frontline workers and clerical staff) dyads	A manufacturing firm owned by a Hong Kong firm in the Guangdong province of China	Self-concept-based theory	Subordinate task performance (negative relationship); organizational citizenship behavior toward the organization (negative relationship)
Chen et al., 2014	601 supervisor-subordinate dyads	27 companies of a Taiwanese conglomerate including manufacturing, construction, finance, media, and service	Social exchange theory	Employee extra-role performance (negative relationship)
Huang et al., 2015	102 independent subsidiaries of a telecommunications corporation	102 counties in China	_	Subsequent revenue growth (positive relationship, low economic munificence; negative relationship, high economic munificence)
Dedahanov et al., 2016	387 employee (highly skilled full-time employees) -leader (entrepreneurs) dyads	Small and medium manufacturing companies in the Republic of Korea	Social exchange theory	Employee voice (negative relationship); creativity (negative relationship)
Chen et al., 2018	309 participants (68.3 percent junior staff and 31.7 percent managers)	Two enterprises located in Beijing mainland of China	Fairness theory; face and favor theory	Procedural fairness perception (positive relationship, high leader renqing orientation; negative relationship, low leader renqing orientation); interactional fairness perception (positive relationship, high leader renqing orientation; negative relationship, low leader renqing orientation); tacit knowledge sharing intention (positive relationship, high leader renqing orientation; negative relationship, low leader renqing orientation)
Tian and Sanchez, 2017	302 employee-supervisor-peer triads	60 technology-based organizations like farm machinery development, computer systems, and electronics in 13 different Chinese provinces	Social identity theory	Employee breakthrough behaviors across cultures (positive relationship)
Wang and Guan, 2018	211 supervisor-subordinate dyads	10 different technology companies located in China	Social identity theory; goal setting theory; achievement goal theory	Learning goal orientation (positive relationship); employee performance (positive relationship)
Duan et al., 2018	324 employees	16 state-owned manufacturing enterprises in China	Theories of motivation and person–environment fit	Employee silence behavior (positive relationship); psychological safety (negative relationship); organization-based self-esteem (negative relationship)
Du et al., 2020	203 employees and their supervisors	39 work teams in China	Exchange theory; intrinsic motivation theory	Employees’ active support for organizational change (negative relationship)
Karakitapoglu-Aygün et al., 2021	409 employees and 72 leaders from 24 organizations in Turkey; 294 full-time employees from 150 organizations in the U.S.	Turkish industries including construction, health, finance, and tourism; U.S. sectors such as healthcare, retail, food, manufacturing, insurance, software development, and IT	—	Quality of communication (positive relationship, in the U.S.); interpersonal interactions (negative relationship, in Turkey)
Rui and Qi, 2021	406 pairs of leaders, supervisors, and employees	95 working teams from 24 companies in China (Jiangsu, Shanghai, Beijing, Zhejiang, Chongqing, and Wuhan), including manufacturing, real estate, food processing, and finance, etc.	Social learning theory; attraction selection attrition theory; social cognition theory; moral liberation theory; social exchange theory	Unethical employee behavior (positive relationship)

Second, although there is a growing interest in the creative deviance domain, researches concentrating on the leadership-level antecedents are still under-developed (Lin et al., 2016). Creative deviance has been widely proved to occur under a range of preconditions, e.g., task autonomy (Masoudnia and Szwejczewski, 2012), information acquisition (Galperin, 2012), human resource management practices (Malik and Lenka, 2019), Machiavellianism (Galperin, 2012), prosocial motivation (Vadera et al., 2013; Shukla and Kark, 2020), psychological capital (Gao et al., 2020), and personal overqualification (Dar and Rahman, 2020). However, there is still a dearth of explorations relating to team leadership. While R&D teams have clear expectations and task assignments based on the development planning of the organizations (Cheung et al., 2016), and the current high-tech workplace is becoming increasingly team-centric (Hülsheger et al., 2009), there remain very little study focusing on creative deviance within a team management context. Therefore, this study utilizes a broad perspective-team authoritarian leadership to creative deviance.

Last but not least, as Appelbaum et al. (2007) noted, creative deviance indicates a fundamental shift away from the notion of conventional, routine innovation, to the idea that R&D staff pursue pioneering, valuable and promising originalities through illegitimate means. In particular, creative deviance is closely related with violation concerns, such as whether the decision to depart from the referent team norms to maximize innovative achievements is warranted at the expense of violating R&D team obligations (Judge et al., 2006; Chan et al., 2013). Since the pro-team essence of creative deviance is implicit, it can be mistaken as a “team misbehavior” unless the value it creates is detected by the leaders (Perry et al., 2016). For these reasons, the engagement in creative deviance, even with good motives, needs to be given serious considerations (Chan et al., 2013; Lin et al., 2016). According to Cheng and Wang (2015), the focus of authoritarian management changes in terms of a continuum ranging from low to high, which signifies that the core issue of concern is not a rigid framework, but undergoes transformations at different extents of leaders’ authority (Zhang and Xie, 2017). Accordingly, it remains uncertain whether team-based authoritarian leadership plays a “facilitator” or “obstacle” role in the constructive deviance for innovation.

We follow LePine et al. (2004); Dooley et al. (2020), and Kronenwett and Rigotti (2020), who considered the incremental occupational stress from dual and opposite aspects: challenge occupational stress and occupational hindrance stress. Challenge occupational stress refers to the constructive pressure supporting personal growth (or even achievement) (Kronenwett and Rigotti, 2020) while hindrance occupational stress is a terrible pressure state to threaten individuals’ development and to constrain personal career progress (Dooley et al., 2020). Specifically, team authoritarian leadership can influence dual occupational stress associated with working circumstance, after which team members would rationally re-examine whether their deviance is appropriate. We also propose that prevention regulatory focus, defined as “fulfilling duties and obligations through responsible behaviors” (Wallace et al., 2013, p. 984), can be elicited by authoritarian management and ultimately, influence employees’ deviant behaviors. Therefore, our paper constructs two independent mediating paths between team authoritarian leadership and creative deviance to verify the mediating effect of dual occupational stress and prevention regulatory focus, respectively. Furthermore, we believe social information processing theory can be an effective explanation to the process in which employees make behavioral decisions when facing authoritarian management. The theory suggests that, when confronted with the specific social environment surrounding them, employees’ perceptions are triggered from these situational cues (Zhu et al., 2019; Boudrias et al., 2021). Studies along this line found that one’s distinct subjective interpretations of various social cues can become a source of individual behaviors (Brown et al., 2017; Zhu et al., 2019; Li et al., 2020). Meanwhile, according to regulatory focus theory, occupational stress factors can trigger the change of individual regulatory focus from progressive, explorative strategies to traditional, unadventurous strategies (Brockner and Higgins, 2001; Brenninkmeijer and Hekkert-Koning, 2015; González-Cruz et al., 2019). As dual occupational stress represents the information appraisal of stressors at work and prevention regulatory focus represents individuals’ attentions to avoiding mismatches and risks, we then we construct a chain mediating path to investigate the internal mechanism of team authoritarian leadership and creative deviance based on social information processing theory and regulatory focus theory.

There is a definite need to investigate the psychologically relevant moderators (i.e., the psychologically boundary conditions regarding when team authoritarian leadership is more or less influential to creative deviance). Researchers have emphasized that creative deviance is a disobedient, well-intentioned, pioneering construct (Criscuolo et al., 2014) that is affected by external environment (Dahling and Gutworth, 2017; Shukla and Kark, 2020) and mental characteristics (Ng and Yam, 2019; Dar and Rahman, 2020). Therefore, conscious judgments from individual mindfulness (Nübold et al., 2019) could influence the occurrence of creative deviance as well as its relationship with authoritarian leadership. Previous study has revealed that employees might form distinct understandings of creative deviance with mindfulness awareness—particularly, when individuals are with high mindfulness trait, the pro-social motivation of creative deviance can be easily valued (Brown and Ryan, 2003; Eisenbeiss and Van Knippenberg, 2015); a mindful subordinate thus can concentrate on the current goals, build a psychological environment for judging the significance of creative deviance, and ultimately, diminish the negative effects of external leadership on creative deviance. Therefore, we explore the moderation effect of individual mindfulness on the relationship between team authoritarian leadership and creative deviance, as well as the chain-mediating effect. That is, mindful subordinates justify the occurrence of creative deviance as a pro-organizational act rationally, although they may face the threat of damage to the well-behaved self-image and severe punishments.

Previous studies obtain numerous valuable conclusions, but there are still some deficiencies. Firstly, these studies only examine the impact effects and processes of authoritarian leadership on employee behaviors based on the dual opposition between bad or good, ignoring the possible curve relationships. Secondly, the impacts of authoritarian leadership is not formed instantly. More comprehensive and systematic impact mechanisms of authoritarian leadership should be considered under the influences of internal individual factors. Lastly, research model variables in line with Chinese national situations is relatively lacking. Chinese employee characteristics such as confucianism need to be included into the future study, so as to provide practical guidance according to local conditions. Taken together, the research issue of this paper is to explore the underlying mechanism model of team authoritarian leadership affecting members’ creative deviance, comprehensively adopting the perceptive tactical chain path research paradigm, and exploring the specific impact of employees’ value concept on the relationship between leadership and their deviant behaviors. This research specifically explains how team authoritarian leadership can finally present the form of inverted-U to promote or inhibit team members’ prosocial deviant innovations by stimulating members’ occupational pressure with inspiring, benignant or threatening, disturbing attributes, and affect these members’ construction deviant behaviors while simultaneously guarding against possible risks from exploratory activities in varying degrees. The study conducted cross-level statistical tests (Dollard and Bakker, 2010) and HLM approach (Zhang et al., 2009) so as to check the measurement models. We used a nonparametric percentile Bootstrap technique with deviation correction (Simar and Wilson, 2007) to measure the chain mediation effect and the moderating effect of individual mindfulness in this chain-mediating path. In addition, the moderating effect of individual mindfulness in the curvilinear relationship between team authoritarian leadership and creative deviance is intuitively reflected by estimating the slopes of the curves and drawing an interaction effect diagram.

## Theory Review and Hypotheses

### Team Authoritarian Leadership and Creative Deviance

Creative deviance is a representative type of constructive deviance that contributes to the benefit of an organization. By nature, creative deviance has dual attributes (Mainemelis, 2010): on the bright side, it is beneficial since it is intended to scoop out the creative potential (Masoudnia and Szwejczewski, 2012; Chowdhury, 2015); on the dark side, however, it is risky since it violates managerial norms or rules, causes administration dilemmas, and challenges the current management system (Criscuolo et al., 2014; Neves and Champion, 2015).

As suggested by Cheng et al. (2004), authoritarian leadership shapes a father-like role in front of employees via the consolidation of traditional culture such as the value system of Confucianism and Legalism, which can, subsequently, form a centralized management structure. Wang and Guan (2018), in a longitudinal examination of 211 supervisor-subordinate dyads who engaged in high-tech activities, also proposed that when leaders exercise authoritarian control to followers, they put forward higher standards for innovation tasks, spur more efforts, and motivate followers to perform better. Low levels of authoritarian leadership facilitate subordinate loyalty to the organization (Karakitapoglu-Aygün et al., 2021), which is a intangible facilitator of creative deviance. These standpoints align with several empirical studies (see Table 1). For instance, Tian and Sanchez (2017), in a study of 60 technology-based organizations, demonstrated that authoritarian leadership is a significant predictor of employee breakthrough behaviors for their demanding and yet selfless stance. More specially, leaders with low levels of authority establish an explicit goal-orientation, and then, encourage altruistic employee behaviors (Aycan, 2006). Meanwhile, prior research has documented that unambiguous goals rationalize individuals’ understanding of deviant behaviors (Vadera et al., 2013). Collectively, when authoritarian leaders set clear goals, team functioning is improved as high levels of concentration and initiative are evidenced among members. According to this logic, when team authoritarian leadership is low, creative deviance is more likely to be viewed as an innovation “lubricant” due to its beneficial attribute.

By contrary, as Jiang et al. (2017) suggested, high levels of authoritarian leadership exerts a series of negative impacts on subordinates’ reactions: it brings high levels of organizational cynicism, heavy workload, role conflict, role ambiguity and psychological contract violation, as well as low levels of job satisfaction. Similarly, Rui and Qi (2021) also demonstrated that leaders with high authority emphasize complete dominance, asymmetric power, and slavish obedience. This may lessen creative deviance as members in R&D teams experience increased shackles, more non-autonomy, and thus they sense greater levels of dissatisfaction. Additionally, when there is absolute and undemocratic domination over team members fulfilling creative responsibilities, it is harder for them to exploit the potential opportunities of breakthrough-type innovation. In this situation, individuals engaging in creative deviance can be considered as “black sheep” because their implicit pro-organization motivation can be neglected. Team authoritarian leadership can diminish members’ creative deviance through reframing this questionable behavior as unacceptable and elevating the worry or anxiety of being punished. As a consequence, creative deviance, which violates leadership and breaks out traditional fences, could be weakened. Taken together, these double lines of reasoning underpin an inverted U-shaped relationship between team authoritarian leadership and creative deviance. That is, the intermediate level of team authoritarian leadership would yield superior creative deviance as compared to low and high levels, separately. Based on these discussions, we therefore hypothesize:

Hypothesis 1: A curvilinear, namely, an inverted U-shaped, relationship is between team authoritarian leadership and creative deviance, such that R&D teams with low or high authoritarian leadership result in less creative deviance than teams with appropriate authoritarian leadership.

### The Mediating Role of Dual Occupational Stress and Prevention Regulatory Focus

As pointed out by LePine et al. (2004); Edwards et al. (2014), and Dooley et al. (2020), dual occupational stress is considered in terms of two separate, theoretically deduced attributes: challenge occupational stress and hindrance occupational stress. This classification conforms to the classic research of LePine et al. (2004), who advocated that a comprehensive assessment of dual occupational stress should capture both conducive occupational stress (i.e., furthering one’s career) and inconducive occupational stress (i.e., hindering one’s current and future development). A process of qualitative change allows people to transfer stress from the challenge attribute to the hindrance attribute, and heightens the threat to their self-realization selves (Ganster and Rosen, 2013).

Researchers further found that one such situational element—leadership style—can significantly impact individuals’ dual occupational stress (Dartey-Baah and Ampofo, 2015; Quade et al., 2019). Dual occupational stress refers to different levels of pressure boosting or interfering with personal capability to achieve valued goals (Rodell and Judge, 2009). Indeed, employees in a less authoritarian environment arise enthusiasm, initiative and agility from challenge occupational stress to make sure that the work expectations can be accomplished splendidly (Yang et al., 2018). In such a situation, the positive aspect of challenge occupational stress, therefore, accelerates creative deviance, since both constructs well respond to the call for team interests. On the contrary, a team operating under highly authoritarian management undergoes strong constraints in job contents and details. Meanwhile, Criscuolo et al. (2014) and Javed et al. (2017) noted that individuals who formerly engaged in well-intentioned deviant conducts might turn to implement cautiously obedient deeds later, for scholars have theorized that leadership-level behaviors can increase subordinates’ hindrance occupational stress by strengthening norms and punishments (Decoster et al., 2014). According to the aforementioned discussion, the dual occupational stress-creative deviance relationship should follow an inverted U-shaped function. Taken these together, this research proposes:

Hypothesis 2a: Dual occupational stress mediates the inverted U-shaped effect of team authoritarian leadership on creative deviance, such that the indirect effect will be positive when dual occupational stress is low and negative when it is high.

Much is already realized about individuals’ prevention regulatory focus and its compulsion, a series of studies have probed the sources, such as intrapersonal pressure, reputational concerns, autonomy support management, and so forth (e.g., Worthy et al., 2009; Pfattheicher, 2015; Lai et al., 2018; Oiknine et al., 2021). Conclusions from numerous researches demonstrated that people with prevention regulatory focus invest time, energy and efforts fully into their duties and obligations and subsequently, beget a sense of security (Worthy et al., 2009; Lai et al., 2018). This prevention-focus state is consistent with an “ought” self—valuing the own responsibility extremely (Wallace et al., 2013; Pfattheicher, 2015), which construes that employees, who view the salient goal as a “non-loss” or “loss,” are likely to utilize high levels of prevention focus when facing superiors with high authoritarian management style.

As discussed before, previous research has revealed that a key premise premise of creative deviance is a path-breaking consciousness (i.e., shaking off objective shackles) for the innovation target (Mainemelis, 2010; Criscuolo et al., 2014). That is, employees’ focus point determines the possibility of subsequent deviance (Criscuolo et al., 2014). More specifically, individually preventive focus serves as a lens which reflects the authority of managers; when team authoritarian leadership is relatively low (e.g., emphasizing the goal-achievement rather than maintaining a control-obedience working environment to the members merely), high performance expectations can effectively activate members’ inner enthusiasm to complete their missions and nourish pro-social deviance beneficial to the team (Lin et al., 2016; Sarpong et al., 2018; Malik and Lenka, 2019). However, as creative deviance stealthily challenges supervisors’ orders, such behavior cannot be forgiven or justified when the team environment is absolutely authoritarian. In this case, prevention-focus individuals are reluctant to perform creative deviance due to their risk aversion and concern about career prospects. This dialectic process reveals the mediation effect of prevention regulatory focus. Namely, prevention regulatory focus mediates the inverted U-shaped association between team authoritarian leadership and creative deviance. By inference, we put forward the following hypothesis:

Hypothesis 2b: Prevention regulatory focus mediates the inverted U-shaped effect of team authoritarian leadership on creative deviance, such that the indirect effect will be positive when prevention regulatory focus is low and negative when it is high.

Social information processing theory (Brown et al., 2017) proposes that individuals get information cues from the external environment where the events occurred and form intentions of subsequent actions. In other words, a person perceives and decodes the information clues so as to further explain and respond to them (Zhu et al., 2019). Specific to the case of creative deviance, individuals will recognize whether to perform it or not, subjective to various environmental cues—typically, leadership factors play remarkable roles in the willingness of creative deviance (Kell, 2018). Based on regulatory focus theory, as Bélanger et al. (2013) mentioned, individuals’ subjective initiative is limited when occupational stress changes from the challenge attribute to the hindrance attribute and strengthens the focus on avoiding negative outcomes and fulfilling the basic job requirements. In conclusion, team authoritarian leadership can advance prevention regulatory focus by stimulating the members’ dual occupational stress, and ultimately influence the individual behavior (creative deviance). Considering the two hypotheses (Hypothesis 2a and 2b) together, we expect that dual occupational stress and prevention regulatory focus can act as a chain mediator between team authoritarian leadership and creative deviance. In light of this, we suggest:

Hypothesis 2c: Dual occupational stress and prevention regulatory focus play a chain-mediating role between team authoritarian leadership and creative deviance; that is, team authoritarian leadership can foster individuals’ prevention regulatory focus by aggravating their dual occupational stress, thereby affect creative deviance in an inverted U-shaped pattern.

### The Moderating Role of Individual Mindfulness

Notwithstanding the fact that exploring the team authoritarian leadership-creative deviance correlation brings meaningful insights into the understanding of authoritarian leadership in R&D teams, there is an omission regarding its psychological moderating roles on such a correlation. In an attempt to open this black box, we aim to test a potential moderator, namely individual mindfulness, and forecasts that the inverted U-shaped association between team authoritarian leadership and creative deviance will be weaker with high levels of mindfulness trait at work. This is because mindfulness represents a compelling force that develops individuals’ open and receptive attitudes (Kiken and Shook, 2011; Reb et al., 2015; Walsh and Arnold, 2020). The key concern of R&D teams is toward pioneering and forward-looking innovation (Hülsheger et al., 2009; Cheung et al., 2016), where team members with mindfulness are more willing to engage in unconventional innovation as they take initiative to execute constructive behaviors (Brown and Ryan, 2003). Mindfulness thus allows employees to stay calm and attentive, be free from distractions, and keep relative calm emotions. Moreover, if two explanations about the current act compete against each other (e.g., accomplishing a job task because of superior directives versus because of pro-organizational motivations), a mindful awareness (e.g., “I understand the essences of superior orders”) can validate the altruistic one (Brown and Ryan, 2003; Dane, 2011; Kiken and Shook, 2011; Hülsheger et al., 2013; Eisenbeiss and Van Knippenberg, 2015; Walsh and Arnold, 2020). Collectively, these consequences impact the levels of creative deviance.

Furthermore, as mindful individuals are more conscious and inclusive (Kiken and Shook, 2011), they are able to overcome fixed thought as well as to pay receptive attentions to their authoritarian leader, and subsequently neglect obvious or potential distractions from the superior (Hülsheger et al., 2013; Jamieson and Tuckey, 2017). Inferentially, when leaders’ authority upgrades to high levels, a highly centralized management system is set up (Chan et al., 2013). Mindful ones need to place less priority on processing authoritarian leaders’ negative messaging, keep creative enthusiasm and accomplish R&D missions attentively (Walsh and Arnold, 2020). As such, any influence derived from team authoritarian leadership on creative deviance would be more difficult to realize when subordinate are mindfulness (Eisenbeiss and Van Knippenberg, 2015). We also believe that our research has a moderated chain-mediating effect. Subordinates who are meditators will respond to pressure events in relaxed ways. Meanwhile, mindful employees are more likely to treat stressors with a more receptive attitude and perspective; thus, mindfulness can effectively reduce the level of stress, anxiety and other emotional problems associated with stress. Therefore, we assume that:

Hypothesis 3a: individual mindfulness moderates the curvilinear relationship between team authoritarian leadership and creative deviance in such a way that the inverted U-shaped manner becomes weaker for those members with intense mindfulness as compared with those with faint mindfulness.
Hypothesis 3b: individual mindfulness moderates the positive effect of team authoritarian leadership on dual occupational stress, thus moderating the chain mediating influence of dual occupational stress and prevention regulatory focus on the curvilinear relationship between team authoritarian leadership and creative creative deviance.

**FIGURE 1:**
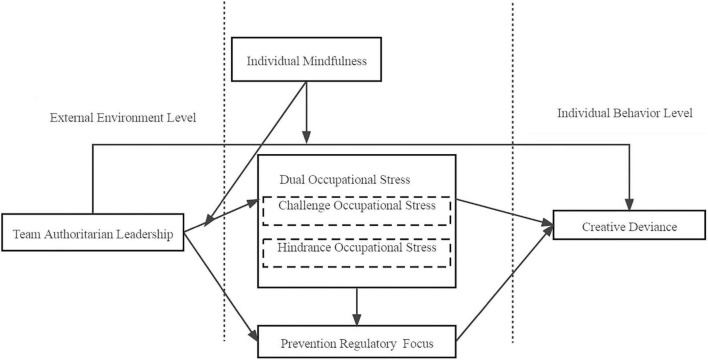
The research conceptual model.

Combining these hypotheses together, we summarize all research variables and hypotheses in one conceptual framework (see Figure 1).

## Materials and Methods

### Research Setting and Procedure

As suggested by Rui and Qi (2021), authoritarian leadership is more influential in the organizations composed of knowledge-based staff. Employees with high levels of professional knowledge and specialties seek autonomy during a R&D process and thus, need more opportunities to get rid of the shackles of traditional management modes. Knowledge employees have the possibility of leveraging their expertise to make leapfrog innovations by taking constructive deviance. This perspective thus whips up the academic debate on the relationship between team authoritarian leadership and creative deviance and extends the externalities of authoritarian leadership theory into R&D teams. Our study chose a Chinese member sample, whereby the conceptualization and evolution of authoritarian leadership is predominantly developed in diverse Chinese cultural settings (see Table 1).

Our research collected data from full-time employees in high-tech companies in electronic information technology, new material technology, new medical technology, resource and environment technology and advanced manufacturing technology located in five provinces in eastern China. We purposely chose our interviewees from such teams as research and development in those types of high and new tech enterprises. The respondents are front-line R&D staff, leadership-contact employees who have considerable interactions with superiors of their team. Since temporal disconnection in a variable correlation analysis can effectively prevent social desirability as well as reverse causation, we conducted this survey in two phases. Specifically, the items for the first phase contained the contents on team authoritarian leadership, individual mindfulness, and control variables; the second phase (1 month later) questionnaire asked all participants regarding their dual occupational stress, prevention regulatory focus and creative deviance. With the assistance from the HR departments, the respondents of these two phases were paired with the last six digits of their cell phone numbers so as to assure coherence. We also gave every respondent a 1–10 yuan random Wechat red envelope after completion in order to ensure a certain response rate. All scales were presented in Chinese after scientific translations and back-translations from the English originals when necessary (Chen and Boore, 2010). We invited two Ph.D. students and a professor majoring in human resources management to inspect the contents of these scales. 5-point Likert scales were used to measure all items but demographic factors, where 1, means “not at all,” and 5, represents “absolutely.” Initially, a pilot testing was conducted with 65 employees from 10 R&D teams. In accordance with the feedback provided, minor corrections were made. We collected 489 completed responses from 90 R&D teams with more than three individuals out of 556 photocopies distributed among 103 teams in the first phase (response rate 87.95%) and 457 completed photocopies in 84 teams in the second phase out of the 489 responses retrieved from the first phase (response rate 93.46%). After pairing each participant in this two-phase survey and carefully inspecting all responses, we deleted the completed questionnaire which were unmatched or unscientific. This finally left us with a valid sample of 433 participants in 82 R&D teams (an average of 5.3 selected members of each team). The specific participant demographics and team characteristics are outlined in Table 2.

**TABLE 2:** Sample characteristics (Ind.: 433; Team: 82).

Characteristics (Ind.)	Indicators	Frequency	Percentage	Characteristics (Team)	Indicators	Frequency	Percentage
**Gender**	Female	112	25.9%	**Size**	5 members and below	4	4.9%
	Male	321	74.1%		6–10 members	13	15.9%
**Highest education**	Bachelor degree	6	1.4%		11–15 members	38	46.3%
	Master degree	235	54.3%		16–20 members	19	23.2%
	Doctoral degree	127	29.3%		21 members and above	8	9.7%
	Postdoctoral degree	65	15.0%	**Industry**	Electronic information technology	12	14.6%
**Team tenure (years)**	≤1	24	5.5%		New material technology	25	30.5%
	(1, 2)	41	9.5%		New medical technology	19	23.2%
	(2, 3)	110	25.4%		Resource and environment technology	15	18.3%
	(3, 4) (4,5)	96 102	22.2% 23.6		Advanced manufacturing technology	11	13.4%
	> 5	60	13.8%				

### Measures

#### Team Authoritarian Leadership

We adapted a nine-item scale verified by Cheng et al. (2004) to a 5-point scale to follow the principle of consistency of all our main measures. Team authoritarian leadership was measured via 9 items containing two theoretically separate properties: “zhuanquan” and “shangyan.” A sample item is “My supervisor determined all decisions in the team whether they are important or not.” This research adopted a cluster aggregation approach to assess the nature of team authoritarian leadership. This aggregation technique is an intrinsically cross-level method that advocates a holistic theoretical and analytical technique to simulating the entirety of the objects rated (Koo and Li, 2016). To justify whether the aggregation is appropriate, we adopted the most common indices of cluster aggregation analysis to explicitly assess the extent to which the same set of members are non-independent on or clustered by a specific team (Biemann et al., 2012). Generally speaking, the intra-class correlations, ICC (s) and the interrater agreement statistic, *r*_wg_, were calculated to identify inter-rater reliability, internal consistency reliability and within-group variance (Koo and Li, 2016). The results revealed that the ICC (1) value of 0.452 demonstrated that response variability at the membership level that is attributed to being part of a group reached a high degree. The ICC (2) value of 0.813 suggested that the group-level mean is reliable. The mean *r*_wg_ value of 0.882 was much larger than the accepted cut-off value of 0.70 (Lance et al., 2006), which implied that there is a high degree of consistency among distinct raters in the selected R&D teams. Given strong supports for the employed measurement approach, thus, this study aggregated these individual-level scores to generate a single variable to reflect team authoritarian leadership. Team ratings were then calculated by summing each of the response from team members divided by the total amount of respondents (Castro, 2002; LeBreton and Senter, 2008). The values of rating ranged from 1.750 to 4.167, where higher scores indicate higher degrees of authoritarian leadership within a team. The reliability coefficient of team authoritarian leadership was 0.838.

#### Creative Deviance

Following Lin et al. (2016), this research assessed the level of creative deviance by requiring the team membership to rate each of his/her deviant behaviors on a following example: “Besides working on ideas that were approved by my supervisor, I also exerted effort in improving the rejected ideas by collecting information and trying again.” It measures the degree to which team subordinates insisted on some of the novel but rejected ideas. The reliability coefficient of creative deviance was 0.787.

#### Dual Occupational Stress

We modified a 14-item questionnaire developed by González-Ramírez and Hernández (2007) to measure dual occupational stress. The duality symbolize the occupational stress from quantitative change to qualitative change. The increasing pressure formation process forms the transformation of opposite attributes. In this vein, the reversal point of stress imply that the attribute interconversion take place with the changeable levels of stress. To emphasize the R&D team context, we adapted the original items in this questionnaire. For example, we changed the item “Have you felt difficulties were piling up so high that you could not overcome them” to “Have you felt difficulties in your team R&D work were piling up so high that you could not overcome them.” It includes reverse consideration questions (a sample item is “Have you dealt successfully with day to day innovations”), these data collected were then reassigned inversely, where the value of 1 or 2 is recoded as 5 or 4, and 3 remains unchanged. The reliability coefficient of this scale was 0.902.

#### Prevention Regulatory Focus

Prevention regulatory focus was assessed using nine items derived from Neubert et al. (2008). These consist of the extent of a subordinate’s concern for costs of loss, failure, or punishment, the prudence to choosing behavior manners, as well as the inclination to avoiding the risk of errors. In accordance with previous research, we included the management contexts of authoritarian leadership in the measurement of prevention regulatory focus (Lin and Johnson, 2018). For example, we changed the item “At work, I am often focused on accomplishing tasks that will support my need for security” in the original prevention regulatory focus scale to “At work, I am often focused on accomplishing tasks that will meet the strict requirements of my authoritarian superior.” The reliability coefficient of this scale was 0.820.

#### Individual Mindfulness

Similar to the study of Lau et al. (2006), this scale contains items on the present-centered, non-elaborative, and non-judgmental aspects of the mindul quality. We added individual mindfulness as the moderator for two reasons. First, research has presented that mindful subordinates’ attention to the authoritarian management mode is intentionally open (Bartlett et al., 2021); second, although creative deviance is risky, members with high individual mindfulness are more willing to take this risk to enhance team benefit (Reb et al., 2015). A sample of 13 items is “I experienced my thoughts more as events in my mind than as a necessarily accurate reflection of the way things ‘really’ are.” The reliability coefficient of this individual mindfulness scale was 0.887.

#### Control Variables

The study controlled gender, highest education, and team tenure at the individual level and size at the team level. Especially, team members’ power distance was also controlled, together with team culture and team strategy for the analysis of this present research. First is power distance, as it has been proposed to be positively related to the emergence of deviant behaviors (Lian et al., 2012) and negatively to the relationship between leader-member exchange and employee self-ratings of constructive actions (Anand et al., 2018). The second control variable is team culture (i.e., the value all members have followed to achieve a specific vision). It was included as it reflects the consensus formed by team members which may influence team deviance (Liao et al., 2004) and authoritarian leadership because team shared values affects the codes of conduct, senses of mission and team consciousness among all members (Joo et al., 2012). Third is team strategy, since the team’s ethical strategy has been demonstrated to mediate the relationship between authoritarian leadership and team identification (Cheng and Wang, 2015). The three Cronbach α coefficients for power distance, team culture and team strategy were 0.803, 0.781, and 0.840, respectively, all above the standard of 0.70. Furthermore, as team culture and team strategy are team-level phenomena, the consistency among these respondents’ ratings was also measured thus proving sufficient intra-team reliability [for team culture, mean *r*_wg_ = 0.901, ICC(1) = 0.427, ICC(2) = 0.797; for team strategy, mean *r*_wg_ = 0.868, ICC(1) = 0.273, ICC(2) = 0.665]. As pointed out by previous research (Liu et al., 2020), when team size is small, a remarkable inter-team difference can be employed as an aggregation criterion in practice. Although team strategy is not up to the best reliability of intra-team average, the result of One-Way ANOVA showed that team strategy in different R&D teams reached significant difference [*F*(81,351) = 2.988, *p* < 0.01].

## Materials and Methods

### Validity Test and Common Method Bias Testing

A disproportion between the number of measurement indicators and sample size could be an issue for our research study that contains large amounts of parameters to be estimated. Hence, following Yang et al.’s (2010) suggestion, we first conducted high-low combinations of factor loadings and correlation analyses. The independent variable (team authoritarian leadership), dependent variable (creative deviance), mediators (dual occupational stress and prevention regulatory focus), and moderator (individual mindfulness) in our present study were all parceled and shortened into 5, 3, 7, 3, and 7 combined items and consequently, reduced the communalities of these variables.

This study performed confirmatory factor analyses estimated by the maximum-likelihood procedure for the five main factors. All model fit indices are shown in Table 3. The hypothesized model was tested by loading the items on their corresponding latent variables. This five-factor model fits the data well: χ^2^(265) = 402.244, *p* < 0.001, root mean square error of approximation (RMSEA) = 0.035, comparative fit index (CFI) = 0.950, non-normed fit index (NNFI) = 0.943, standardized root mean square residual (SRMR) = 0.044. Indeed, our hypothesized model was superior to the other alternatives. For instance, the four-factor model combining dual occupational stress and prevention regulatory focus fits significantly worse than the five-factor model, using the chi-square difference test [Δχ^2^(4) = 474.965, *p* < 0.001]. We also examined the three-factor model [e.g., M3: Δχ^2^(7) = 910.700, *p* < 0.001], the two-factor model [Δχ^2^(9) = 1069.684, *p* < 0.001], and the single-factor model [Δχ^2^(10) = 1232.081, *p* < 0.001]. In order to prevent the sample size from interfering with the above judgment conclusions, we compared the variations of Akaike information criterion (ΔAICs) according to the suggestions of Burnham and David (2002). The AIC value of the five factor model (522.244) is the minimum, and ΔAICs (135.288–1212.081) are all greater than 10, which reveals that the time-delay data in this study no longer support other competitive models.

**TABLE 3:** Confirmatory factor analysis.

Models	χ^2^	df	Δχ^2^	RMSEA	CFI	NNFI	AIC	ΔAIC	SRMR
Five-factor model (M8): T_AL_, I_M_, D_OS_, P_RF_, C_D_	402.244	265	—	0.035	0.950	0.943	522.244	—	0.044
Four-factor model^1^ (M7): T_AL_ + I_M_, D_OS_, P_RF_, C_D_	545.532	269	(143.288)***	0.049	0.899	0.887	657.532	135.288	0.050
Four-factor model^2^ (M6): T_AL_ + D_OS_, I_M_, P_RF_, C_D_	802.696	269	(400.452)***	0.068	0.804	0.782	914.696	392.452	0.063
Four-factor model^3^ (M5) : T_AL_, I_M_, D_OS_ + P_RF_, C_D_	877.209	269	(474.965)***	0.072	0.777	0.751	989.209	466.965	0.067
Three-factor model^1^ (M4): T_AL_ + I_M_ + C_D_, D_OS_, P_R_	1045.840	272	(643.596)***	0.081	0.716	0.687	1151.840	629.596	0.072
Three-factor model^2^ (M3): T_*AL*_, I_*M*_, D_*OS*_ + P_*RF*_ + C_*D*_	1312.944	272	(910.700)***	0.094	0.618	0.579	1418.944	896.700	0.082
Two-factor model (M2): T_AL_ + D_OS_ + P_RF_ + C_D_, I_M_	1471.928	274	(1069.684)***	0.101	0.560	0.519	1573.928	1051.684	0.087
Single-factor model (M1): T_AL_ + I_M_ + D_OS_ + P_RF_ + C_D_	1634.325	275	(1232.081)***	0.107	0.501	0.456	1734.325	1212.081	0.092
Null model	3024.300	300	(2622.056)***	0.145	0	0	3524.300	2732.056	0.241
Judgment criteria (Hu and Bentler, 1999)				<0.050>0.900>0.900>AIC (saturated model)					< 0.080

*N (R&D team members) = 433.*

***p < 0.001.

*T_AL_, team authoritarian leadership; I_M_, individual mindfulness; D_OS_, dual occupational stress; P_RF_, prevention regulatory focus; C_D_, creative deviance; similarly hereinafter. +, the combination of two variables into one. χ^2^, chi-square; df, degree of freedom; Δχ^2^, the χ^2^ result compared with the χ2 value of the hypothesized 5-factor model; RMSEA, root mean square error of approximation; NNFI, non-normed fit index; AIC, Akaike information criterion; ΔAIC, the AIC result compared with the AIC value of the hypothesized 5-factor model; and SRMR, standardized root mean square residual.*

Furthermore, specifically, the factor loadings (i.e., the factor-measurement item correlations) for were strong (i.e., Std. Estimate > 0.70, *p* < 0.001) for team authoritarian leadership items, 0.667–0.909; creative deviance items, 0.771–0.823; dual occupational stress items, 0.648–0.815; prevention regulatory focus items, 0.714–0.799; individual mindfulness items, 0.742–0.801. With regards to the average variances extracted (AVE) of all scales, each of them (0.510, 0.553, 0.570, 0.604, and 0.532 for team authoritarian leadership, creative deviance, dual occupational stress, prevention regulatory focus, and individual mindfulness) reached the proposed level, i.e., greater than the acceptable minimum of 0.50 (Koufteros, 1999), indicating that the measures had ideal convergent validity, together with the adequate discriminant validity of the default model.

In order to check the existence and magnitude of common method variance, we adopted a multitrait-multimethod (MTMM) developed by Podsakoff et al. (2003). Results of the test of a controlled six-factor model [χ^2^ = 400.593, df = 264, χ2/df = 1.517, RMSEA = 0.035, CFI = 0.950, Tucker-Lewis index (TLI) = 0.943; SRMR = 0.039, Δχ^2^/Δdf = 1.651, ns] showed that the more complicated model, in which all items loaded both on their respective construct as well as a a latent method factor, did not show a significantly better fit yet (ΔRMSEA, ΔCFI, ΔTLI, ΔSRMR are all less than 0.02). Meanwhile, we adopted a principal component with a varimax rotation analysis. Comparing with the variance explanation of the eigenvalues greater than 1-factor (61.14%), Harman’s single-factor test yielded the first factor explaining only 29.76% of the total variance. Therefore, we can legitimately concluded that there was not significant common method bias in the present measurement.

### Descriptive Statistics

Table 4 quantifies the means, standard deviations (SDs), and zero-order correlations for each of the constructs. For example, as illustrated, team authoritarian leadership at the team level is negatively and significantly correlated to dual creative deviance (γ = –0.257, *p* < 0.01); dual occupational stress is positively and significantly associated with team authoritarian leadership (γ = 0.363, *p* < 0.01) at the individual level, and prevention regulatory focus (γ = 0.318, *p* < 0.01), but is negatively and significantly with creative deviance (γ = –0.233, *p* < 0.01); prevention regulatory focus has a significantly negative association with creative deviance (γ = –0.219, *p* < 0.01); and meanwhile, individual mindfulness has a significantly negative correlation to dual occupational stress (γ = –0.227, *p* < 0.01), but a significantly positive correlation with creative deviance (γ = 0.208, *p* < 0.01), which provided preliminary evidence to support the hypotheses in the present study.

**TABLE 4:** Descriptive statistics and correlations.

Objects	Variables	*M*	*SD*	1	2	3	4	5	6	7	8
	(1) Gender	0.741	0.386								
	(2) Highest education	1.580	0.753	0.069							
	(3) Team tenure	2.903	1.048	–0.038	–0.024						
**Ind. level**	(4) Power distance	2.748	1.162	0.093	–0.131*	0.045					
	(5) T_AL_^a^	2.152	0.827	0.008	–0.011	–0.152*	0.107				
	(6) I_M_	2.229	0.971	–0.086	0.075	–0.018	0.066	0.021			
	(7) D_OS_	2.548	0.955	–0.092	–0.083	–0.067	0.079	0.363**	–0.227**		
	(8) P_RF_	2.306	0.938	–0.101	–0.095	–0.049	0.088	0.339**	–0.176*	0.318**	
	(9) C_D_	3.065	0.964	0.090	0.164*	0.037	–0.185*	–0.257**	0.208*	–0.233**	–0.219**
	(1) Size	2.573	0.981								
	(2) Team culture	2.720	1.034	0.075							
	(3) Team strategy	2.575	1.007	0.089	0.184*						
	(4) T_AL_^b^	2.085	0.772	0.129*	0.161*	0.097					

*N (Ind.) = 433, N (Team) = 82. Gender (Ind. level): 0 = Female, 1 = Male; Highest education ((Ind. level): 0 = Bachelor degree, 1 = Master degree, 2 = Doctoral degree, 3 = Postdoctoral degree; Length in the team (Ind. level): 0 represents “Year ≤ 1,” 1 represents “1 < Year ≤ 2,” 2 represents “2 < Year ≤ 3,” 3 represents “3 < Year ≤ 4,” 4 represents “4 < Year ≤ 5,” 5 represents “Year > 5”; Size (Team level): 0 = 5 members and below, 1 = 6–10 members, 2 = 11–15 members, 3 = 16–20 members, 4 = 21 members and above; Industry (Team level): 0 = Electronic information technology, 1 = New material technology, 2 = New medical technology, 3 = Resource and environment technology, 4 = Advanced manufacturing technology.*

*p < 0.05, **p < 0.01.

*Team authoritarian leadership were under statistics at both individual level and team level, and the results are represented by T_AL_^a^ and T_AL_^b^ separately.*

### Hypothesis Testing

**FIGURE 2:**
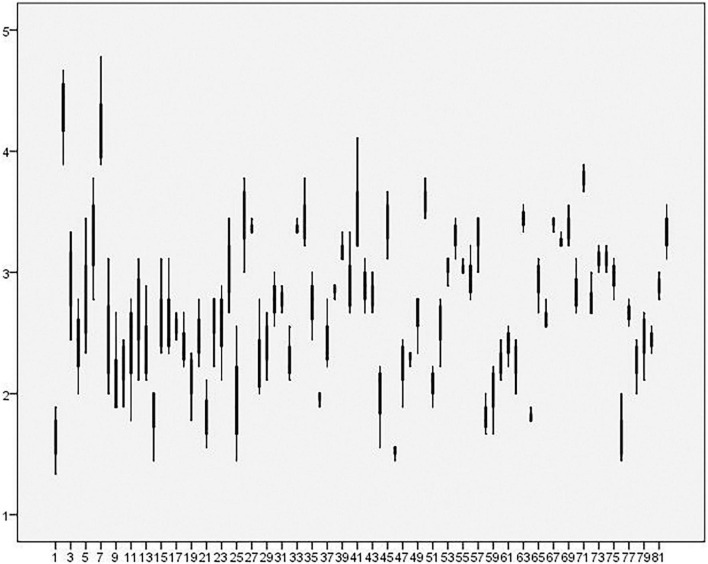
The box-and-whisker plot of member creative deviance for 82 independent R&D teams (Sorting by the team identifier).

Figure 2, a box-and-whisker plot for each sample team, visually depicts the significant variations of creative deviance at individual level as well as team level. This study employed cross-level statistical tests (Dollard and Bakker, 2010; Preacher, 2015; Tremblay, 2017). Led by the procedure delineated in Zhang et al. (2009)’s research, in hierarchical linear modeling, the centralized group mean of each individual-level predictor, dual occupational stress and prevention regulatory focus, was implemented in this study; these centralized variables as control variables were then included into the intercept equations. Table 5 depicts the results of the multilevel analyses.

**TABLE 5:** HLM analysis results.

Fixed-effect	D_OS_	P_RF_	C_D_			
	Model 1	Model 2	Model 3	Model 4	Model 5	Model 6
	**Coefficient (*SE*)**					
Intercept term	3.014*** (0.075)	2.358*** (0.051)	3.040*** (0.066)	3.838*** (0.074)	2.204*** (0.047)	2.469*** (0.050)
**Level-1 control variables**						
Gender	–0.058 (0.068)	–0.045 (0.097)	0.021 (0.045)	–0.010 (0.018)	–0.029 (0.040)	–0.026 (0.042)
Highest education	–0.074 (0.079)	–0.018 (0.056)	0.047 (0.063)	0.082 (0.093)	0.062 (0.098)	0.055 (0.068)
Team tenure	–0.025 (0.028)	–0.021 (0.037)	0.053 (0.048)	0.088 (0.131)	0.059 (0.074)	0.057 (0.151)
Power distance	0.103+ (0.055)	0.069 (0.121)	–0.047 (0.061)	–0.013 (0.045)	–0.051 (0.082)	–0.029 (0.058)
**Level-1 predictors**						
D_OS_					1.062*** (0.173)	
D_OS_^2^					–0.287** (0.080)	
P_RF_						0.901*** (0.145)
P_RF_^2^						–0.265** (0.072)
I_M_				–0.621** (0.175)		
**Level-2 control variables**						
Size	0.021 (0.043)	0.009 (0.095)	–0.011 (0.029)	–0.009 (0.046)	–0.007 (0.009)	–0.015 (0.024)
Team culture	–0.065 (0.111)	–0.037 (0.084)	0.049 (0.108)	0.062 (0.069)	0.058 (0.093)	0.043 (0.075)
Team strategy	0.037 (0.078)	0.026 (0.042)	–0.032 (0.053)	–0.029 (0.051)	–0.033 (0.057)	–0.024 (0.049)
Group mean of D_OS_					1.870*** (0.366)	
Group mean of D_OS_^2^					–0.625** (0.181)	
Group mean of P_RF_						1.142*** (0.227)
Group mean of P_RF_^2^						–0.586** (0.168)
Group mean of I_M_				–0.640** (0.182)		
Group mean of T_*AL*_^2^ × I_M_				–0.174* (0.060)		
**Level-2 predictors**						
T_AL_	0.352** (0.112)	0.304** (0.085)	1.269*** (0.249)	0.535** (0.150)		
T_AL_^2^			–0.326** (0.095)	–0.090+ (0.049)		
**Cross-level interaction item**						
T_AL_^2^ × I_M_				–0.129* (0.047)		
**Random effect**						
Inter-group variation	0.128*** (101.031)	0.153*** (112.446)	0.195*** (116.873)	0.078** (79.218)	0.192*** (122.012)	0.186*** (121.420)
Slope variance				0.058 (67.949)	0.024 (67.114)	0.043 (70.886)
Intra-group variation	0.762	0.804	0.872	0.739	0.783	0.885
–2 Log likelihood	750.408	767.603	795.044	751.968	773.892	783.427

*N (R&D team members) = 433.*

***p < 0.001, **p < 0.01, *p < 0.05, +p < 0.1.

*The χ2 values of random effects are shown in brackets.*

#### Null Models

A substantial inter-group variation of each dependent variable in a null model is an important prerequisite. For dual occupational stress, we found that the inter-group variation (τ00) and intra-group variation (σ^2^) were 0.149 (χ^2^ = 110.239, *p* < 0.001) and 0.764, and the inter-group variation accounted for 16.3% of the total variation; for prevention regulatory focus, the τ00 and σ^2^ value was 0.186 (χ^2^ = 121.250, *p* < 0.001) and 0.792, and the τ00 value accounted for 19.0% of the total variation; for creative deviance, we found the inter-group variation (0.251, χ^2^ = 137.464, *p* < 0.001) accounted for 22.6% of the total variation. These results provided compelling evidence that the legitimacy of our multilevel analyses is acceptable.

#### Mediating Effect of Dual Occupational Stress and Prevention Regulatory Focus

As shown in Table 5, the square of team authoritarian leadership is significantly as well as negatively associated with creative deviance (γ = –0.326, *p* < 0.01, Model 3), supporting Hypothesis 1. The square of dual occupational stress is significantly and negatively related to creative deviance (γ = –0.287, *p* < 0.01, Model 5). The effect of team authoritarian leadership on dual occupational stress is significant and positive (γ = 0.352, *p* < 0.01, Model 1), which is consistent with Hypothesis 2a. In addition, the relationship between the squared term of prevention regulatory focus and creative deviance is negative (γ = –0.265, *p* < 0.01, Model 6). Team authoritarian leadership has an significantly positive effect on prevention regulatory focus (γ = 0.304, *p* < 0.01, Model 2) and consistent with our Hypothesis 2b.

To better illustrate the chain-mediating role of dual occupational stress and prevention regulatory focus, we utilized a nonparametric percentile Bootstrap technique with deviation correction following Simar and Wilson’s (2007) approach—with MPLUS 7.4 (Muthn, L. K. and Muthn, B. O., Los Angeles, CA, United States). The complete model linked up with a chain mediation has a high fitness (χ^2^ = 175.182, df = 129, RMSEA = 0.031, SRMR = 0.038, CFI = 0.954, NNFI = 0.949). More specifically, Table 6 suggests that when the branched mediation path follows “Team authoritarian leadership→Dual occupational stress→Creative deviance,” team authoritarian leadership positively affects dual occupational stress; then, the effect of dual occupational stress on creative deviance presents inverted U-shaped; that is to say, the necessary condition of an independent mediating effect is met (β = –0.097, 95% CI [–0.134, –0.057]). By the same token, when the branched mediation path is “Team authoritarian leadership→Prevention regulatory focus→Creative deviance,” team authoritarian leadership has a significantly positive effect on prevention regulatory focus; prevention regulatory focus has a significantly inverted-U effect on creative deviance; this meant that, prevention regulatory focus can independently mediate the relationship between team authoritarian leadership and creative deviance (β = –0.070, 95% CI [–0.105, –0.033]). When the chain terms entered the relationship, its chain-mediating effect on creative deviance is still significant but is slightly weakened (β = –0.038, 95% CI [–0.042, –0.031]). Percentages of mediating effects were used to quantify the effect quantity of each mediation path and to lay the foundation for estimating the total effect of authoritarian leadership on team members’ creative deviance. Therefore, the proposed Hypothesis 2a, 2b, and 2c were supported.

**TABLE 6:** Model-Fit test and mediating effects test.

Model	Graphic description	χ2	df	χ2/df		RMSEA	SRMR	CFI	NNFI
Complete model	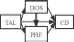	175.182	129	1.358		0.031	0.038	0.954	0.949

**Effects**		**Estimated values**			**95% LLCI**		**95% ULCI**	**Proportion**	

Direct effect of T_AL_ on C_D_		–0.154** (Inverted U)			–0.219		–0.087	42.90%	
Decomposition of indirect mediating effects									
H2b: T_AL_→D_OS_→C_D_ (Independent mediating path 1)		–0.097** (Inverted U)			–0.134		–0.057	27.02%	
H3b: T_AL_→P_RF_→C_D_ (Independent mediating path 2)		–0.070* (Inverted U)			–0.105		–0.033	19.50%	
H4: T_AL_→D_OS_→P_RF_→C_D_ (Independent mediating path 3)		–0.038* (Inverted U)			–0.052		–0.021	10.58%	
Total effect of T_AL_ on C_D_		–0.359*** (Inverted U)			–0.528		–0.186	100.00%	

*N (R&D team members) = 433.*

***p < 0.001, **p < 0.01, *p < 0.05.

*Bootstrap based on repeating sampling 20,000 times.*

#### Moderating Effect of Individual Mindfulness

We explored the processes by which team authoritarian leadership and individual mindfulness interact to influence the proposed chain-mediating effect and creative deviance, separately (Table 7). We first tested this moderated chain-mediating effect. We used a mean benchmark, where members with a value of individual mindfulness equal to 1.258 (Mean – 1 SD) defined as a low level and the value equal to 3.200 (Mean + 1 SD) was a high level. Accordingly, the chain mediating effect of dual occupational stress and prevention regulatory focus on an inverted-U relationship existing between team authoritarian leadership and creative deviance is –0.055 (*p* < 0.05, 95% CI [–0.068, –0.039]) with a low level of individual mindfulness, whereas it is –0.018 (ns, 95% CI [–0.026, –0.008]) when individual mindfulness is high. Hence, the chain mediating effect is no longer significant. Meanwhile, there is a significant difference between the effect values of the chain-mediating path (Estimate = –0.037, *p* < 0.05, 95% CI [–0.049, –0.022]). Therefore, Hypothesis 3a received supported.

**TABLE 7:** Cross-level moderated chain-mediating effect analysis.

Moderators	Chain-mediating path: T_AL_→D_OS_→P_RF_→C_D_			
	Phases			Total chain-mediating effects
	Phase I: T_AL_→D_OS_	Phase II: D_OS_→P_RF_	Phase III: P_RF_→C_D_	
Low I_M_ (Mean – 1 *SD* = 1.258)	0.426*** (0.369, 0.481)	0.370*** (0.320, 0.416)	–0.348*** (Inverted U) (–0.384, –0.308)	–0.055* (Inverted U) (–0.068, –0.039)
High I_M_ (Mean + 1 SD = 3.200)	0.231** (0.194, 0.265)	0.306** (0.277, 0.331)	–0.247** (Inverted U) (–0.293, –0.199)	–0.018 (Inverted U) (–0.026, –0.008)
Differences	0.195** (0.166, 0.222)	0.064* (0.045, 0.080)	–0.101** (Inverted U) (–0.145, –0.054)	–0.037* (Inverted U) (–0.049, –0.022)

*N (R&D team members) = 433.*

***p < 0.001, **p < 0.01, *p < 0.05.

*Bootstrap based on repeating sampling 20,000 times.*

**FIGURE 3:**
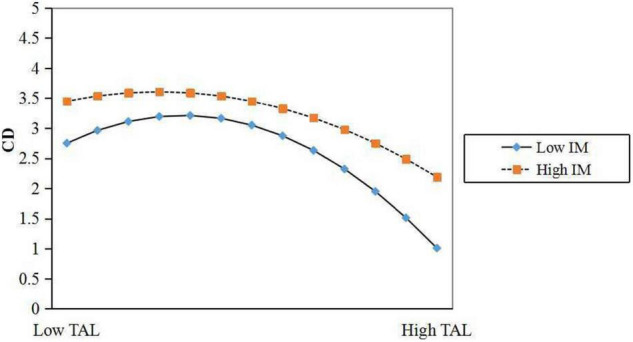
The moderating role of individual mindfulness on the relationship between team authoritarian leadership and creative deviance.

As previously revealed in Table 5, comparing with Model 3, the direct inverted U-shaped effect of team authoritarian leadership on creative deviance is still significant but evidently reduced (γ = –0.090, *p* < 0.1, Model 4). The interactive effect is statistically negative on creative deviance (γ = –0.129, *p* < 0.05, Model 4). This means that individual mindfulness can negatively moderate the relationship between team authoritarian leadership on creative deviance. We next plotted the relationship between team authoritarian leadership and creative deviance as moderated by individual mindfulness according to the steps recommended by Mai et al. (2021). To do this, a slope estimation was created. Figure 3 graphically illustrates the inverted-U relationship between team authoritarian leadership on creative deviance with low individual mindfulness vs. high mindfulness. The radians and inflection points symbolize the moderating effects. We see that an inverted-U relationship is stronger with low individual mindfulness, when compared to the individual characteristic of high mindfulness. Therefore, Hypothesis 3b was fully supported in this research.

## Discussion

By integrating the concepts from team authoritarian leadership and creative deviance literature, this current study sheds light on our understanding of whether and when team authoritarian leadership is positively or negatively related to creative deviance. More specifically, this current research therefore demonstrated that an inverted-U association between team authoritarian leadership and creative deviance holds up in Chinese R&D teams, thus providing cogent work in the antecedents and conditions of creative deviance. Especially, our cross-level approach furnished empirical evidence that team authoritarian leadership impacts members’ creative deviance through the chain mediation effect of dual occupational stress and prevention regulatory focus. This research also demonstrated that the trait of individual mindfulness moderates the relationship between team authoritarian leadership and creative deviance as well as the chain-mediating effect; where the inverted-U association is indeed weaker with high levels of individual mindfulness. Now, this research further discusses the theoretical contributions and practical implications of these findings, along with some limitations of the present work and possible directions for future study. Overall, our study not only enriches the empirical literature on creative deviance, but also sheds light on how to practice authoritarian leadership in the workplace realistically.

### Theoretical Contributions

First of all, authoritarian leadership has been mostly shown to be adverse for employees’ beneficial behaviors in practice as well as in the extant literature (Chen and Kao, 2009; Zhang et al., 2011; Chan et al., 2013; Dedahanov et al., 2016; Duan et al., 2018; Spagnoli et al., 2020; Rui and Qi, 2021). Although these studies have confirmed the disadvantages of authoritarian leadership, there are still several disagreements and controversies surrounding it (Aycan, 2006; Huang et al., 2015; Chen et al., 2018). By joining a handful of researchers in the field of the positive effects of authoritarian leadership (e.g., Cheng et al., 2004; Wang and Guan, 2018; Karakitapoglu-Aygün et al., 2021), we further confirm that authoritarian leadership can play a driving role in facilitating constructive team behaviors. Despite previous study pointing out that creative deviance seems related to superior management (Chan et al., 2013; Lin et al., 2016), it has rarely been conceptualized as a leadership-related decision issue, and little is known about how different types of leadership styles systematically shape creative deviance. Specifically, this study extended this line of research and linked team authoritarian leadership with creative deviance (defined in terms of its nature of an ethical trade-off between deontological and pragmatistic methodology; Appelbaum et al., 2007). Our results explicitly addressed the ethical nature of creative deviance and expanded its antecedents and mechanisms from a leadership perspective. Moreover, our research also responds to the appeal of Cheng et al. (2004) by clarifying the process by which authoritarian leadership influences subordinates’ behaviors.

At the same time, we also shed a light on the specific psychological mechanisms through which team authoritarian leadership is related to creative deviance. Our findings confirmed that under different attributes of dual occupational stress, team authoritarian leadership has opposite effects on creative deviance. This result of the investigation is consistent with the theory on the dynamic essence of dual occupational stress. On the one hand, as Dooley et al. (2020) noted, dual occupational stress is not a static, but an accumulative and a qualitative change process, wherein challenge stress and hindrance stress alternate as the degree of pressure changes. Specifically, R&D team members, whose focus is on the strategy generation to overcome difficulties with the challenge attribute of occupational stress, proactively participate in constructive innovation process. It thus provides a positive environment to nourish creative deviance. Our results extend the occupational stress theory by identifying challenge stress as an underlying motivation to encourage radical innovations. Our study also contributes to the understanding of prevention regulatory focus in the workplace. Previous study regarded the attention to organizational interests as the major source of employees’ good deeds (such as ethical behaviors and constructive behaviors, etc.) (Mainemelis, 2010; Criscuolo et al., 2014). Our work suggests that prevention regulatory focus can impact creative deviance by its nature of avoiding harm. Moreover, this finding also supports the proposition proposed by Pfattheicher and Sassenrath (2014) who asserted that risk-avoidance strategies generated in a prevention-focused orientation pay more attention to fulfilling the basic work needs. Last but not least, we provided empirical evidence for the chain-mediating effect of dual occupational stress and prevention regulatory focus as one route through which creative deviance increases or decreases.

Another fold of the significant theoretical contributions is that the current study provides new insights into an important boundary condition of authoritarian leadership effects. We investigated and demonstrated that the individual trait of mindfulness (considered in terms of the awareness to accept each thought, feeling, or sensation as it is and to keep a present-centered intellect) moderates the team authoritarian leadership-creative deviance association and the chain mediating effect; such relationship and influence are weaker with high levels of mindfulness. This finding agrees well with previous researches that suggested that individual mindfulness fosters a non-elaborative and non-judgmental pattern of cognitive reactivity. For example, Reb et al. (2015) suggested that employees with mindfulness experience less emotional distress, lower vulnerability, and higher spiritual well-being. Walsh and Arnold (2020) proposed that individual mindfulness exerts positive impacts on one’s curiosity, acceptance, and openness to reality as mindfulness inherently advocates greater inclusiveness. Such high levels of mindfulness shown by individuals helps to cultivate an intentional self-regulation of attention, which could sequentially be beneficial and evolve into effective results. Therefore, as demonstrated in this current study, R&D team members who are mindful, show increased acceptance, higher levels of openness, and a greater sense of concentration.

### Practical Implications

As a matter of fact, creative deviance has become a new normalcy for R&D teams (Criscuolo et al., 2014). It dynamically seeks an optimal balance between maintaining teams’ innovative capability and violating managerial orders when ingenious ideas are rejected (Criscuolo et al., 2014). Creative deviance becomes increasingly significant in businesses today, that is, it can be advantageous to improve innovation performance (Chowdhury, 2015). For example, Galperin (2012) have suggested that R&D staff engaging in nonconforming innovations resulting from unsanctioned bottom-up pioneering initiatives, sometimes bring about revolutionary ideas of potentially great benefit. Thus, based on our study findings, we would like to enable team managers and leaders to focus on the formation of creative deviance in practice. Most notably, the inverted-U team authoritarian leadership–creative deviance relation indicates that authoritarian leadership can be a useful way to promote team innovation. This suggests that R&D team managers seeking to motivate members should specify the overall objectives within the teams and take steps to encourage the members to undertake R&D roles actively and provide them with adequate opportunities to give scope for their professional abilities.

Moreover, this research provides a benchmark with individual-environment relation perspective to reveal subordinates’ violation of a managerial order, in order to determine the extent to which dual occupational stress and prevention regulatory focus are reinforcing or relieving the formation of creative deviance as a chain influence process. Therefore, on the one hand, managers cannot directly encourage subordinates to engage in creative deviance given its particularity and complexity. They should build or support a goal-oriented but not repressive working climate (Vadera et al., 2013). For instance, low authoritarian leaders can indirectly promote employees’ creative deviance through facilitating occupational challenge stress by setting clear career goals. On the other hand, some individuals’ basic self regulatory orientations, such as prevention regulatory focus, can influence creative deviance by navigating their valued standards and raising the sensitivity to the presence of negative outcomes (Wallace et al., 2013). Thus, managers should employ proper authoritarian means to stimulate creative deviance and utilize the amplification effect of prevention regulatory focus in the loss-averse process. Besides, this study emphasizes the need for individual members to cultivate a mindful awareness particularly in an authoritarian environment in order to minimize negative impacts. Gong et al. (2009) also argued that the effects of leadership have a close connection with individual sensations, thoughts, as well as emotions. Combining these arguments with our results, R&D teams should shape mindful team value to enhance members’ open awareness, attenuate adverse effects of authoritarian leadership, and facilitate team benefits concurrently.

### Limitations and Directions for Future Research

As is the case for any research, we would be evasive if we did not acknowledge some limitations related to this current study that are worthy of being addressed in future. First, since the measurements used in our research were taken from the same source and all self-reported, there could be common method bias influencing the effects. We have adopted several methods to minimize its possible effects. This study ensured all responses’ confidentiality by requiring participants to answer questionnaires anonymously, while the entire data were collected in two phases and some were analyzed by a cluster aggregation method. We also adopted a MTMM approach and a cross-level design to detect common method bias. As such, common method bias was mitigated to some extent. In addition, the sample of our experimental study consisted of 82 R&D teams, however, there is still a need to revalidate our hypotheses from more multiple sources so as to achieve more robust statistical power.

Second, while the definition of creative deviance (measured in terms of order-violation innovations and covert innovations) is multidimensional in nature, it does not take every possible aspect into consideration, e.g., effectiveness of innovation. In other words, there can be more consequences of team authoritarian leadership that have not been accounted for, especially outcomes from a team-level perspective, e.g., comprehensive advantages of team innovation. Therefore, future research can make meaningful contributions by examining the relationship between team authoritarian leadership and its outcomes from a wider variety of research backgrounds. Furthermore, we also cannot exclude other reasonable theoretical explanations for these confirmed hypotheses. One such explanation could be that team authoritarian leadership and creative deviance might share a same common origin. For instance, external competition of enterprises could be another reason that team authoritarian leadership has an influence on creative deviance. Conclusions of past researches have revealed that intense industry competition environment can promote the occurrence of authoritarian leadership (Farh and Cheng, 2000) and constructive deviance (Galperin, 2012). Considering the fact that the aforesaid possibilities could contaminate this research, we employed a two-phase method to collect data and followed a logical reasoning with rigor to prove the rationality of our results. Even so, future study should still pay attention to those alternative explanations more cautiously.

Third, an obvious finding of our investigation, regarding how team authoritarian leadership influences creative deviance with the increase of degree, shows the double-edged effects of team authoritarian leadership. Its impacts are likely to be affected by cultural contexts (e.g., high or low collectivism); as well as work settings (e.g., environmental uncertainties). Scholars, like Tian and Sanchez (2017), have sought more attention to be paid to explaining whether its theoretical models change along with distinct contextual effects. Unfortunately, the design of this current research did not directly examine these factors that could stimulate or impede the external effects of team authoritarian leadership. It thus would be a promising research direction. We call for future research to provide a more integral explanations of the boundary conditions, particularly for external environment-related moderators. Examples like industrial complexity thus open up new horizons for future studies. Moreover, the potential individual perceptual indicators should also be examined considering the effects of authoritarian leadership are cognitive evaluation processes in nature. This would serve as another promising research direction. Our research suggests future studies on authoritarian leadership take an interdiscipline perspective. For instance, study with a neuroscience design that captures event-related potential (ERP) of authoritarian leadership, to explore how it guides or drives throughout the brain information processing, is another fruitful avenue for future research.

Fourth, creative deviance is an environmentally sensitive and emerging creative behavior. We tested its conceptual model only in high-tech industries since the measure of creative deviance that we adopted reflected some characteristics of these enterprises. However, team members working in other industries such as service might engage in different types of creative deviance from that of technical employees. Therefore, this study encourages future research to develop an optimum scale to measure creative deviance in a wider scope. Creative deviance, as a new innovatiaon pattern that has been demonstrated to facilitate innovation performance in research institutions. However, we do not assert that creative deviance is a panacea for all innovative woes. It is argued that there may be several circumstances where creative deviance causes penalties, e.g., wastage of resources and deteriorated leader member exchange (Shukla and Kark, 2020). Meanwhile, many researchers have also pointed out that the practice of creative deviance simultaneously includes positive and negative components, so it is essential to explore whether it benefits R&D teams. In light of this, study concerning whether creative deviance is beneficial when R&D team members experience varying degrees of authoritarian leadership should thus be another interesting research avenue.

## Data Availability Statement

The original contributions presented in the study are included in the article/supplementary material, further inquiries can be directed to the corresponding author/s.

## Ethics Statement

Written informed consent was obtained from the individual(s) for the publication of any potentially identifiable images or data included in this article.

## Author Contributions

JX contributed to conducting analysis and editing the final manuscript as submitted. Y-ZL generated the study idea with the theoretical development of the research. D-QZ was responsible for the structure and content, and revised all versions of the manuscript. J-ZL collected all the data as well as did the data analysis. All authors contributed to the article and approved the submitted version.

## Conflict of Interest

The authors declare that the research was conducted in the absence of any commercial or financial relationships that could be construed as a potential conflict of interest.

## Publisher’s Note

All claims expressed in this article are solely those of the authors and do not necessarily represent those of their affiliated organizations, or those of the publisher, the editors and the reviewers. Any product that may be evaluated in this article, or claim that may be made by its manufacturer, is not guaranteed or endorsed by the publisher.
